# COVID‐19 and inequities in colorectal and cervical cancer screening and diagnosis in Washington State

**DOI:** 10.1002/cam4.4655

**Published:** 2022-03-18

**Authors:** Ofer Amram, Solmaz Amiri, Jeanne Robison, Chaya Mangel Pflugeisen, Pablo Monsivais

**Affiliations:** ^1^ Elson S. Floyd College of Medicine Washington State University Spokane Washington USA; ^2^ Department of Nutrition and Exercise Physiology, Elson S. Floyd College of Medicine Washington State University Spokane Washington USA; ^3^ Paul G. Allen School for Global Animal Health Washington State University Pullman Washington USA; ^4^ MultiCare Institute for Research & Innovation Tacoma Washington USA; ^5^ MultiCare Deaconess Cancer & Blood Specialty Centers Spokane Washington Spokane Washington USA

**Keywords:** COVID‐19, colorectal cancer, cervical cancer

## Abstract

**Introduction:**

Studies have shown that cancer screenings dropped dramatically following the onset of the coronavirus diseases 2019 (COVID‐19) pandemic. In this study, we examined differences in rates of cervical and colorectal cancer (CRC) screening and diagnosis indicators before and during the first year of the COVID‐19 pandemic.

**Methodology:**

We used retrospective data from a large healthcare system in Washington State. Targeted screening data included completed cancer screenings for both CRC (colonoscopy) and cervical cancer (Papanicolaou test (Pap test)). We analyzed and compared the rate of uptake of colorectal (colonoscopies) and cervical cancer (Pap) screenings done pre‐COVID‐19 (April 1, 2019–March 31, 2020) and during the pandemic (April 1, 2020–March 31, 2021).

**Results:**

A total of 26,081 (12.7%) patients underwent colonoscopies in the pre‐COVID‐19 period, compared to only 15,708 (7.4%) patients during the pandemic, showing a 39.8% decrease. A total of 238 patients were referred to medical oncology for CRC compared to only 155 patients during the first year of the pandemic, a reduction of 34%. In the pre‐COVID‐19 period, 22,395 (10.7%) women were administered PAP tests compared to 20,455 (9.6%) women during the pandemic, for a 7.4% reduction. period 1780 women were referred to colposcopy, compared to only 1680 patients during the pandemic, for a 4.3% reduction.

**Conclusion:**

Interruption in screening and subsequent delay in diagnosis during the pandemic will likely lead to later‐stage diagnoses for both CRC and cervical cancer, which is known to result in decreased survival.

**Impact:**

The results emphasize the need to prioritize cancer screening, particularly for those at higher risk.

## Graphical abstract

Studies are showing that COVID‐19 had an impact on access to colorectal and cervical cancer screening. However, only limited number of studies focused on inequities in colorectal and cervical cancer screening during the pandemic and little has been reported on the impact on diagnosis. This study shows lower screening and diagnosis during COVID‐19, particularly for patients residing in rural areas.

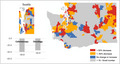

What is already known on this subject?

Studies are showing that coronavirus diseases 2019 (COVID‐19) had an impact on access to colorectal and cervical cancer screening. However, only a limited number of studies focused on inequities in colorectal and cervical cancer screening during the pandemic, and little has been reported on the impact on diagnosis.

What does this study add?

This study shows lower screening and diagnosis during COVID‐19, particularly for patients residing in rural areas.

## 1 INTRODUCTION

One documented outcome of the coronavirus diseases 2019 (COVID‐19) pandemic is the disruption of secondary preventive care, including cancer screening.^1^, ^2^, ^3^ Studies from the United States and other countries have shown that cancer screenings dropped dramatically during the COVID‐19 pandemic.^4^, ^5^ A recently published US study found that screening and diagnostic mammogram fell by 58% and 38%, respectively, in the 20 weeks after March 11.^1^ Other studies have shown that delayed screening will increase the likelihood of a late cancer diagnosis, potentially leading to higher mortality rates. For instance, a UK modeling study estimated that delayed and missed breast screenings will likely increase breast cancer deaths by 7.9–9.6%.^6^

Cancer screening aims to detect malignancy at an early stage and increases the likelihood of curing patients before the onset of symptoms, at a time when cancer treatment is highly effective. This is true for all cancers, including cervical and colorectal cancer (CRC).^7^ Data from the United States suggest that advances in screening technology and screening programs, achieved in recent decades, have resulted in a substantial decrease in cancer mortality and morbidity.^7^ For example, the early identification of precursors like cervical intra‐epithelial neoplasia for cervical cancer led to a 60% decrease in the incidence of this cancer over time.^7^, ^8^ However, there is evidence of inequities in access to screening for both CRC and cervical cancer in the United States, where minority, low socioeconomic status (SES), and rural populations have poorer access to these lifesaving services.^2^, ^9^, ^10^ There is also ample evidence that inequities in access to screening lead to disparities in health outcomes in terms of mortality and morbidity.^11^, ^12^

There have been substantial disruptions in cancer screening and other health care services during the COVID‐19 pandemic.^2^, ^13^, ^14^ Social distancing and lockdown measures implemented in Washington State and nationally forced many health services to delay screening services for at least a two‐month period beginning in March 2020, and this was followed by further disruptions throughout the pandemic. In this study, we used data from a large Washington State clinical network to study rates of CRC and cervical cancer screening and diagnosis before and during the first year of the COVID‐19 pandemic. We examined changes in screening and diagnosis overall and among sociodemographically‐defined subpopulations.

## 2 METHODS

### 2.1 Data

We retrieved retrospective data from MultiCare, one of the largest non‐profit healthcare systems in Washington State. This health care delivery network includes over 230 primary care, specialty care, and urgent care clinics, as well as eight hospitals across Washington State. Targeted screening data included completed cancer screenings for both CRC (colonoscopy) and cervical cancer (Pap test). We did not include CRC home tests like FIT, gFOBT, or FIT DNA as any worrisome results will require a timely colonoscopy. We also analyzed data points that suggested new cancer diagnoses detected by these screenings, including new referrals to medical oncology for patients with a diagnosis of CRC and procedure codes identifying women who underwent colposcopy following a screening pap smear. The inclusion criteria focused on patients aged 18 and over who had at least one cancer screening (colonoscopy or PAP test), as well as women who were referred for a colposcopy procedure in the year prior to the pandemic (April 1, 2019–March 31, 2020). In addition, we examined the number of new medical oncology referrals for colorectal cancer within this health system during the first year of the pandemic (April 1, 2020–March 31, 2021). The study's protocol was approved by the Institutional Review Board of MultiCare, the data holder.

### 2.2 Measures and statistical analysis

Sociodemographic data included patients' race and ethnicity (non‐Hispanic White, non‐Hispanic Black, non‐Hispanic Asian, non‐Hispanic Native Hawaiian and Other Pacific Islander (NHOPI), non‐Hispanic American Indian and Alaska natives (AIAN), multiracial, Hispanics), insurance type (Commercial, Government, Medicare, Medicaid and self‐pay), age (18–49, 50–69, and 70 and over), gender (male/female—only for colonoscopy), and zip code of residence. Rural–urban commuting area (RUCA) codes differentiated between urban versus rural residence based on the zip code of residence.^15^ Frequency analysis and chi‐square tests were performed to assess differences between the two time periods. We used R for Statistical analyses and ESRI ArcGIS for mapping.^16^, ^17^

## 3 RESULTS

The number of patients served by MultiCare (measured as unique patients who completed an encounter with a primary care within the health system) remained stable during the pandemic with 193,174 patients in 2018, 214,305 in 2019, and 211,554 in 2020. This indicates that any change in screening during the pandemic is not due to the change in total patients served by MultiCare.

### 3.1 Colorectal cancer

#### 3.1.1 Colonoscopies

**FIGURE 1:**
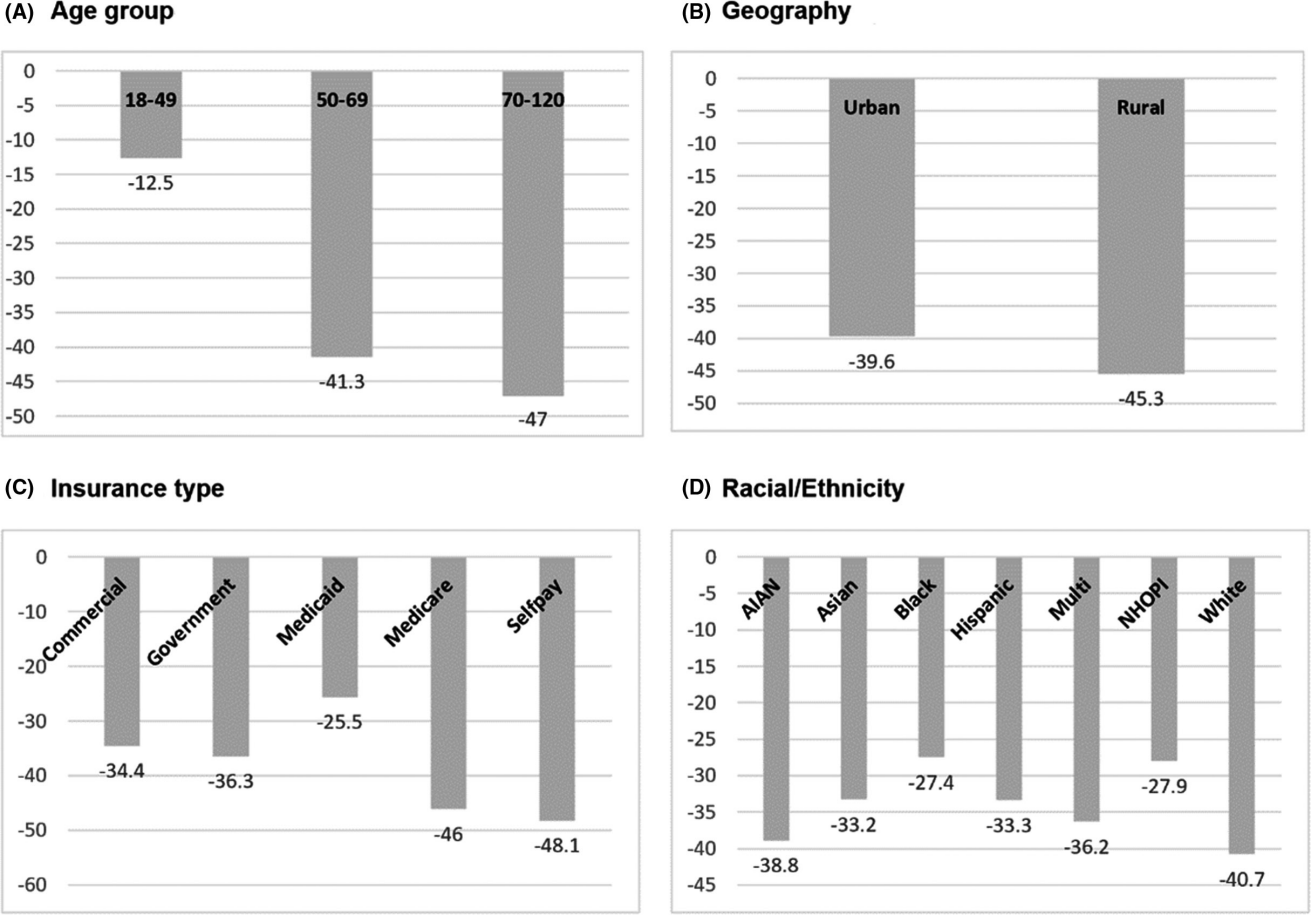
Reductions in colonoscopies pre and during COVID‐19 by age (panel a), by rural/urban residence (panel b), by type of insurance coverage (panel c), and by race/ethnicity (panel d). *p*‐values from Chi‐square tests comparing pre and during COVID‐19 values

There were 26,081 (12.7% of total patients in MultiCare) patients who underwent colonoscopies in the pre‐COVID‐19 period, compared to only 15,708 (7.4% of total patients in MultiCare) patients during year one of the pandemics, showing a 39.8% overall decrease (Table 1). The decline in screenings varied among racial groups, with Whites and AIANs showing a 40% and 38.8% decline, respectively, while blacks and NHOPIs showing the smallest reductions of 27.4% and 27.9%, respectively (*p* < 0.001, Figure 1). In terms of insurance, patients who self‐paid or were on Medicare experienced the largest reduction in screenings (48.1% and 46%, respectively), while those with commercial insurance or Medicaid showed smaller reductions (34.4% and 25.5%, respectively – *p* < 0.001). In addition, patients aged 70 and over experienced a 47% reduction in screenings, the most of any other age group in the study (*p* < 0.001).

**TABLE 1:** Characteristics of patients undergoing colonoscopy pre and during COVID‐19

Variable	Pre pandemic colonoscopy	During pandemic colonoscopy (*n* = 15,708)	*p*‐value
	(*n* = 26,081)		
	*n* (%)	*n* (%)	
Age group			
<50	2885 (11.8)	2523 (17)	*p* < 0.001
50–69	15,763 (64.7)	9248 (62.3)	
70+	7433 (30.5)	3937 (26.5)	
Racial category			
Non‐Hispanic White	22,322 (91.7)	13,235 (89.2)	<0.001
Asian	786 (3.2)	525 (3.5)	
Black	781 (3.2)	567 (3.8)	
NHOPI	122 (0.5)	88 (0.6)	
AIAN	160 (0.7)	98 (0.7)	
Hispanic of any race	839 (3.4)	560 (3.8)	
Mixed race	809 (3.3)	516 (3.5)	
Geography			
Urban	25,256 (96.8)	15,258 (97.1)	0.1343
Rural	823 (3.2)	450 (2.9)	
Insurance			
Commercial	11,446 (47)	7514 (50.7)	*p* < 0.001
Government	551 (2.3)	351 (2.4)	
Medicaid	282 (1.2)	210 (1.4)	
Medicare	11,533 (47.4)	6223 (41.9)	
Selfpay	285 (1.2)	148 (1)	
Gender			
Male	14,071 (57.8)	8478 (57.1)	0.739
Female	12,009 (49.3)	7230 (48.7)	

*Note*: Commercial insurance was administered by a non‐governmental entity. Government insurance was administered by a governmental entity. Self‐pay patients paid out‐of‐pocket and were largely uninsured and low SES.

Abbreviations: AIAN, American Indian/Alaska Natives; NHOPI, Native Hawaiian and Other Pacific Islander; Rural, based on Rural–Urban Commuting Area (RUCA classification 1–3); Urban, based on Rural–Urban Commuting Area classification 4–10.

Finally, no substantial differences were found between patients residing in rural (45.3%) and urban (39.6%) zip codes (*p* = 0.1343) or between males and females (*p* = 0.7394, Not shown in Figure 1).

#### 3.1.2 Referrals to medical oncology

**FIGURE 2:**
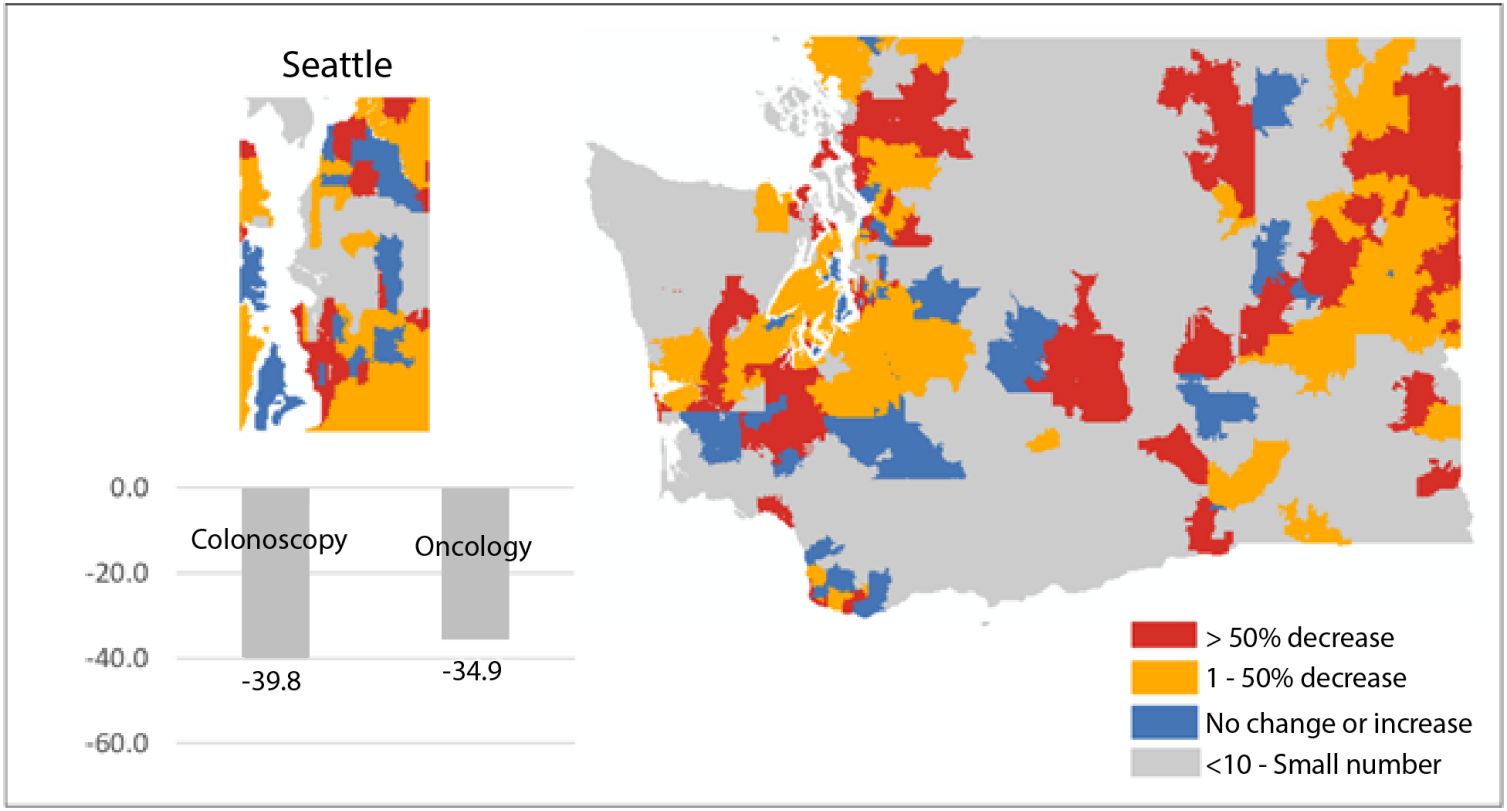
Percentage decrease in colonoscopies and medical oncology referrals pre and during the COVID‐19 periods. Zip code level map highlighting differences in percentage change in colonoscopies pre and during the COVID‐19 periods in WA State

There were 238 patients who were referred medical oncology in the pre‐COVID‐19 period, compared to only 155 patients in the 12 months following the onset of the US pandemic, for a reduction of 34.0%. This is slightly lower than the 39.8% reduction in screenings (Figure 2).

### 3.2 Cervical cancer

#### 3.2.1 PAP test

**FIGURE 3:**
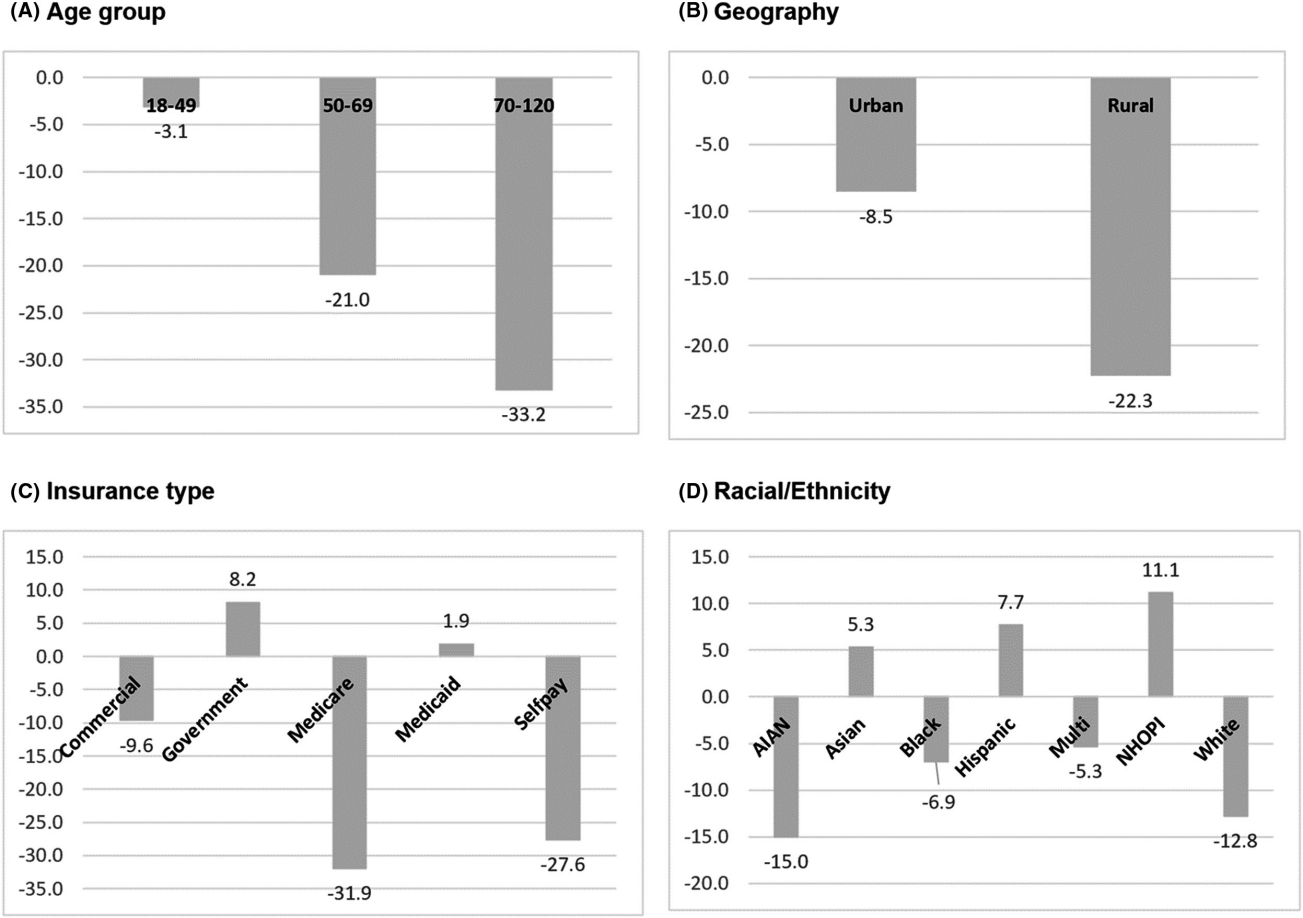
Reductions in PAP tests pre and during COVID‐19 by age (panel a), by rural/urban residence (panel b), by type of insurance coverage (panel c), and by race/ethnicity (panel d). *p*‐values from Chi‐square tests comparing pre and during COVID‐19 values

There were 22,395 women (10.7% of total patients in MultiCare) who were administered PAP tests in the pre‐COVID‐19 period, compared to 20,455 women (9.6% of total patients in MultiCare) during year one of the pandemic, for a 7.4% reduction (Table 2). Declines in PAP tests varied by racial group, with AIANs and Whites showing 15% and 12.8% reductions in screening, respectively. In contrast NHOPIs, Asian and Hispanic women showed relative increases in screenings during this period, of 11.1% and 8%, respectively (*p* < 0.001). Women residing in rural areas showed much larger reductions in screenings at 22.3% versus only 8.5% for their urban counterparts (*p* < 0.001). In terms of insurance, women who are on Medicare and those who self‐pay for insurance experienced the largest reduction in screenings (31.9% and 27.6%) while those insured by Medicare, or a government agency showed a slight proportional increase in PAP test screenings (*p* < 0.001) (Figure 3).

**TABLE 2:** Characteristics of women undergoing PAP tests pre and during COVID‐19 (*n* = 42,850)

Variable	Pre pandemic PAP tests (*n* = 22,395)	During pandemic PAP tests (*n* = 20,455)	*p*‐value
	*n* (%)	*n* (%)	
Age group			
<50	15,684 (71.9)	15,199 (79.4)	<0.001
50–64	6316 (28.9)	4992 (26.1)	
65+	395 (1.8)	264 (1.4)	
Racial category			
Non‐Hispanic White	15,732 (72.1)	13,722 (71.7)	<0.001
Asian	1411 (6.5)	1486 (7.8)	
Black	1402 (6.4)	1305 (6.8)	
NHOPI	350 (1.6)	389 (2)	
AIAN	120 (0.5)	102 (0.5)	
Hispanic of any race	1448 (6.6)	1560 (8.2)	
Mixed race	1751 (8)	1658 (8.7)	
Geography			
Urban	22,180 (99)	20,291 (99.1)	<0.001
Rural	211 (1)	164 (0.9)	
Insurance			
Commercial	15,747 (72.2)	14,232 (74.4)	<0.001
Government	552 (2.5)	597 (3.1)	
Medicaid	2487 (11.4)	2534 (13.2)	
Medicare	1525 (7)	1038 (5.4)	
Selfpay	551 (2.5)	399 (2.1)	

*Note*: Commercial insurance was administered by a non‐governmental entity. Government insurance was administered by a governmental entity. Self‐pay patients paid out‐of‐pocket and were largely uninsured and low SES.

Abbreviations: AIAN, American Indian/Alaska Natives; NHOPI, Native Hawaiian or Pacific Islander; Rural, based on Rural–Urban Commuting Area (RUCA classification 1–3); Urban, based on Rural–Urban Commuting Area classification 4–10.

#### 3.2.2 Referrals to colposcopies

**FIGURE 4:**
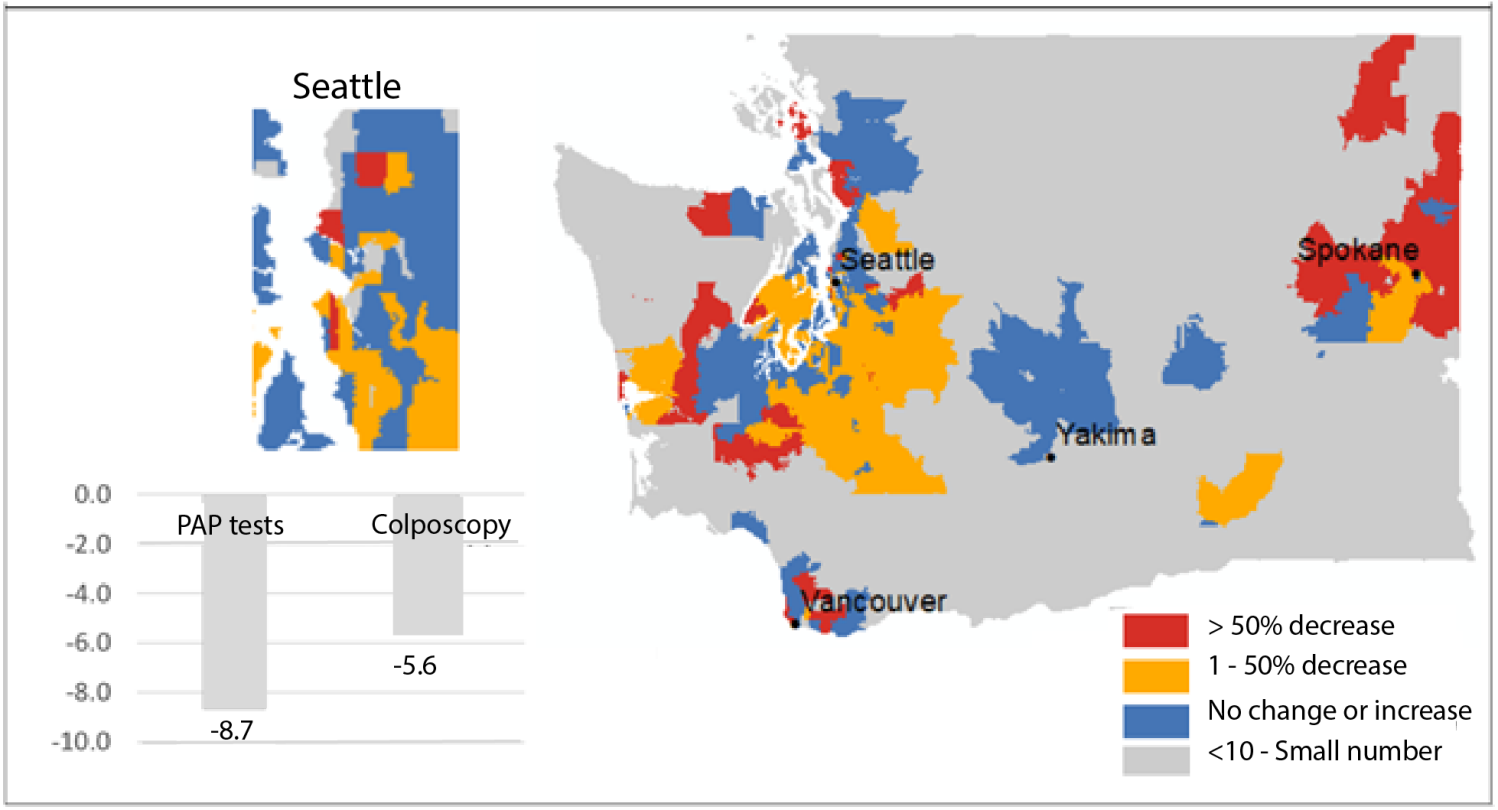
Percentage reduction in PAP tests and colposcopies pre and during the COVID‐19 periods. Zip code level map highlighting differences in percentage changes in PAP tests pre and during the COVID‐19 periods in WA state

There were 1780 women who were referred to colposcopy in the pre‐COVID‐19 period, compared to only 1680 patients during the first 12 months of the pandemic, for a 4.3% reduction. This is slightly lower than the 7.4% reduction in screenings during COVID‐19 (Figure 4).

## 4 DISCUSSION

Using data from a large, state‐wide health care system in Washington State, our study found declines in screenings for both CRC and cervical cancer during the first year of the pandemic. The decline in colorectal cancer screening was substantially larger compared to the reduction in screening for cervical cancer screening, suggesting that the impact of the pandemic on the early detection of colon cancer may be greater than the effect on the early detection of cervical cancer. It is also possible that interruption in screening and subsequent delay in diagnosis during the pandemic could lead to later‐stage diagnoses for both CRC and cervical cancer which is known to result in decreased survival.

Several studies have observed inequities in screening for cancer during the pandemic, including CRC and cervical cancer.^2^, ^9^, ^13^ Here, we observed stark inequalities in reductions in cancer screenings at the onset of the pandemic, with older and rural patients experiencing a substantially larger drop in screenings compared to their younger and urban counterparts. These differences are likely because older populations will tend to skip screening and avoid going to the clinic to minimize exposure to COVID‐19. The relative decrease in screening for those residing in rural areas is not surprising and is likely the result of much poorer access to health services. Screening utilization based on race and ethnicity also differed markedly. However, unlike in other studies, our data show that Whites were less likely than non‐Whites to access screening for both CRC and cervical cancer during this first year of the pandemic.^18^, ^19^ There is a need to further investigate why Whites showed sharp declines in screening and whether the trend continued into the second year of the pandemic. Screening patterns by race and ethnicity may be related to the large urban–rural inequities we also observed, with patients from rural communities much less likely to participate in screenings than urban patients.^2^ Rural areas of the state are less racially diverse than urban areas, with a higher percentage identifying as White.

Finally, the results of this study may also shed light on what we can anticipate in future years in terms of the later stage at diagnosis for both CRC and cervical cancer due to the reductions or delays in screenings. Our data suggest a substantial decrease in patients with a diagnosis of CRC being referred to a medical oncologist during the first year of the pandemic. It is unlikely that this decline in medical oncology referrals was due to improved screening and earlier stages of diagnoses, as we saw a decline in screenings during this time, unfortunately, this may represent a decrease in the identification of individuals with stage II or stage III disease due to the pandemic. Further analysis will be needed to determine if this has resulted in a higher percentage of stage IV colorectal diagnoses and ultimately worse survival data for the years that follow the pandemic.

We also observed a moderate decrease in referrals for colposcopies for patients who were screened for cervical cancer. The purpose of colposcopy is to identify and treat pre‐cancerous and malignant cells from the cervix. This decline in colposcopies may lead to excess future diagnoses of advanced‐stage cervical cancers.

Modeling studies have predicted increases in late‐stage cancer diagnoses during the pandemic. Our study contributes to the body of research on this important issue by quantifying missed diagnoses for both CRC and cervical cancer and how they may impact cancer treatment and outcomes in the coming years.^2^, ^20^, ^21^ It is, therefore, crucial to address this problem by maintaining screening services during a pandemic and scheduling patients who are due for a screening as soon as possible.

Differences in the delivery of screenings between CRC and cervical cancer may also explain the differences in the magnitude of the drop in screenings between the two cancers during COVID‐19.^22^ Papanicolaou test **(**Pap) smears are typically done in a primary care setting and therefore, are accessible throughout WA state providing more opportunities to patients to access screening. In contrast, colonoscopy is typically performed by a gastroenterologist and often requires anesthesiology services. This screening modality required patients to potentially incur a greater cost, agree to greater risk of exposure to Covid 19, and had the potential for greater reduction in services when hospital systems were overwhelmed by spikes in hospitalizations due to the pandemic.^23^ However, the decline in both types of screenings may have resulted in worse outcomes for patients with new colorectal or cervical cancers diagnosed in the state, as referrals to both colposcopy and to medical oncology declined within the system during this time.

### 4.1 Study limitations

This study has several limitations. First, the demographic makeup of this clinical population is less racially diverse and is more affluent (based on insurance type) than WA State's overall population. Second, the data used is from a single health care system and consequently does not necessarily reflect trends in health care utilization in other clinical networks. However, given that MultiCare is one of the largest care providers in the state, the substantial drop in screenings we report is not likely to be explained by a drop in the underlying population base or its eligibility, or even a shift to different providers.

## 5 CONCLUSION

This study examined the change in CRC and cervical cancer screening and diagnosis pre and during COVID‐19. We found a reduction in both screening and diagnosis for both cancers. Future studies should assess whether the reduction will lead to an increase in late‐stage diagnosis or access mortality as a result of the delay.

## CONFLICT OF INTEREST

The authors have no conflict of interest to declare.

## AUTHOR CONTRIBUTIONS

**OA**—Concept, writing, analysis, and interpretation of data; **SA**—analysis and interpretation of data; **JR**—Writing and study design; **CP**—Data acquisition and analysis; **PM**—Concept and interpretation of data.

## ETHICS APPROVAL

The study's protocol was approved by the Institutional Review Board of MultiCare.

## CONSENT TO PARTICIPATE

Not Applicable.

## CONSENT FOR PUBLICATION

Not Applicable.

## AVAILABILITY OF DATA AND MATERIAL

The dataset used in this study is not publicly available as it contains proprietary information that the authors acquired through a license from MultiCare. Information on how to obtain the data is available from the corresponding author on request.

## CODE AVAILABILITY

Not Applicable.

## Data Availability

The dataset used in this study is not publicly available as it contains proprietary information that the authors acquired through a license from MultiCare. Information on how to obtain the data is available from the corresponding author on request.
